# Predictors of two-year mortality in patients with dementia with Lewy bodies

**DOI:** 10.55730/1300-0144.5593

**Published:** 2022-12-13

**Authors:** Neslihan KAYAHAN SATIŞ, Mehmet İlkin NAHARCI

**Affiliations:** Department of Geriatrics, University of Health Sciences, Gülhane Training and Research Hospital, Ankara, Turkey

**Keywords:** Lewy body dementia, prognosis, mortality, malnutrition

## Abstract

**Background/aim:**

Data on adverse prognostic factors for mortality in patients with dementia with Lewy bodies (DLB) are limited. The objective of this study was to evaluate two-year mortality predictors in patients with DLB.

**Materials and methods:**

Individuals aged ≥ 60 years with a diagnosis of DLB, followed by a tertiary-referral geriatric outpatient clinic from 2006 to 2021, were assessed retrospectively using medical or patient records. The mortality status of the patients in the second year after diagnosis was determined. Demographic and clinical characteristics were reviewed to determine their impact on mortality prediction.

**Results:**

A total of 108 patients with DLB participated in this study. The mean age was 78.9 ± 6.6 years, and 49.1% were females. At the end of the two-year follow-up, 23 patients (21.3%) died and 85 patients (78.7%) were still alive. Malnutrition, and cognitive and functional impairments were significantly more common in the mortality group. Age, female sex, functional impairment, moderate-to-severe clinical dementia rating, and malnutrition were associated with an increased mortality risk. On the multivariable analysis, malnutrition (HR = 5.00; 95% CI: 1.64–15.24; p: 0.005) was the only independent predictor of two-year-mortality.

**Conclusion:**

Patients with DLB had an unfavorable survival outcomes. Approaches to prevent malnutrition can improve prognosis and reduce early mortality in this vulnerable group. However, further studies are needed to determine mortality risk factors in this population.

## 1. Introduction

Dementia is a geriatric syndrome characterized by progressive deterioration in cognitive function and capacity to live independently [1]. Dementia with Lewy bodies (DLB), associated with α-synuclein accumulation in the brain stem, basal ganglia, and cortex, is the most common type of degenerative dementia after Alzheimer’s disease (AD) [2]. The prevalence of DLB in all dementia cases in the over 65-years-old population ranged from 3.0% to 26.3% [3,4].

The prognosis of DLB is poorer than that of AD and other subtypes of dementia due to increased morbidity and faster decline in cognition and physical functioning, accompanied by behavioral problems and parkinsonism [5–12]. A few studies reported an increase in the risk of death, ranging from 35% to 88%, in patients with DLB than in those with AD [9,13,14]. Additionally, the average survival time after the diagnosis of dementia in patients with DLB is shorter than in AD [8,13,14].

A few studies have investigated mortality risk factors among community-dwelling patients with DLB. A cohort study with a small sample size identified that each 1-year increase in age at diagnosis increased the risk of mortality by approximately 2.5-fold in individuals with DLB [15]. Amnestic impairment during the course of DLB is associated with a poorer survival rate than nonamnestic impairment [7]. Using data from the National Alzheimer’s Coordinating Center participants with dementia, a recent study has shown that worse baseline cognitive status and more depression significantly affect long-term mortality in DLB [16]. Although studies have shown that biomarkers, brain imaging, and psychocognitive performance are predictive of mortality, geriatric syndromes and comorbidity burden have not yet been examined [7,16–18].

Considering the association between comorbidities and adverse health outcomes in older individuals, there is a need for further investigation of chronic conditions accompanying LBD at advanced ages. Expanding knowledge of the course and prognosis of DLB by identifying mortality modifiers could guide future research to improve the level of care provided by caregivers and institutions. Therefore, this study aimed to investigate two-year all-cause mortality in patients with DLB and the disease characteristics associated with unfavorable survival.

## 2. Methods

### 2.1. Study design

**Figure 1:**
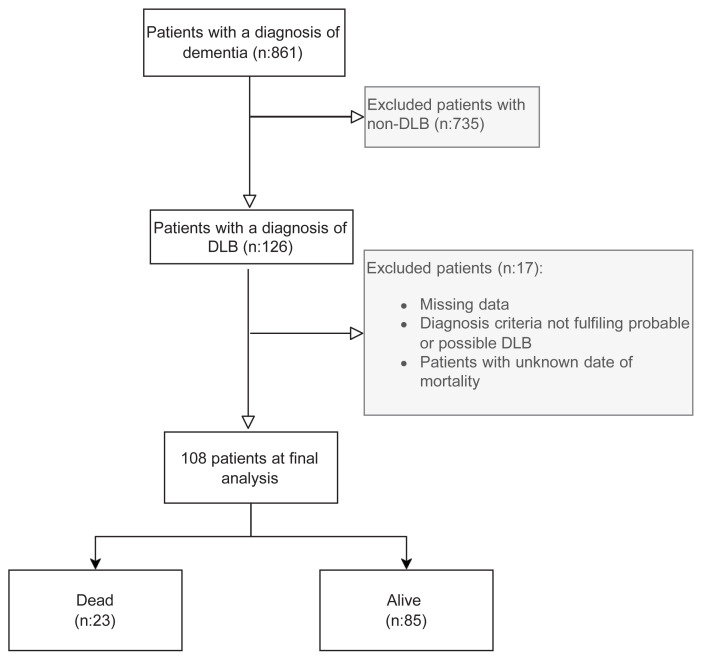
Flow chart of the study design. *Non-DLB include Alzheimer dementia, vascular dementia, frontotemporal dementia, Parkinson dementia and overlap of those dementia types. ** Other than depression.

Individuals aged ≥ 60 years with a diagnosis of dementia (n: 861) admitted to a referral geriatric outpatient clinic between 2006 and 2021 were retrospectively evaluated using their medical records and/or patient charts. Patients with clinically diagnosed DLB who had at least two-year follow-up were enrolled in this study. Mortality status at the end of the two-year was noted. Participants with an unknown date of mortality, diagnosis of other dementias, missing data, or criteria not fulfilling probable or possible DLB were excluded (Figure 1).

This study was performed in accordance with the Declaration of Helsinki, and the local ethics committee approved it (2021/1648-290).

### 2.2. Diagnosis of dementia and DLB

The routine clinical assessment of dementia includes reviewing patient history, physical and mental status, laboratory tests, and radiological examinations (computed tomography or magnetic resonance imaging) for the required cases (i.e. unexplained clinical deterioration). Screening tests for the diagnosis of dementia were conducted using The Mini-Mental State Examination (MMSE) to evaluate the mental status in outpatient clinical settings.

The ‘Diagnostic and Statistical Manual of Mental Disorders Definition’ (DSM) – 4 and DSM – 5 were used for the diagnosis and classification of dementia subtypes via consensus in a panel of two experienced geriatricians. The final diagnosis of probable and possible DLB depended on the international consensus criteria developed and revised by the Consortium on DLB [2,19]. The severity of DLB at diagnosis was determined using the Clinical Dementia Rating (CDR) scale and graded as 1 (mild), 2 (moderate), and 3 (severe) [20].

### 2.3. Patient and disease characteristics

Sociodemographic variables, including age, sex, education, marital status, living status, current smoking status, and alcohol use, were recorded. The Charlson comorbidity index (CCI) was used to measure disease burden [21].

Of geriatric syndromes, functional impairment, urinary incontinence, malnutrition, polypharmacy, and fall history were evaluated at the baseline. The Barthel index (range 0–100) was used to assess functional impairment, as defined by <90 points [22]. Urinary incontinence was determined by a positive answer to questions regarding urine leakage. The Mini Nutritional Assessment-Short Form (MNA-SF) (range 0–14) was used for nutritional status, and a score ≤7 denoted malnutrition [23]. Polypharmacy was defined as concomitant use of five or more drugs. A history of falls in the previous year was also identified.

The DLB-related both core (visual hallucinations, cognitive fluctuations, parkinsonism, and REM sleep behavior disorder) and supportive (delusions, other than visual hallucinations, postural hypotension, and neuroleptic hypersensitivity) clinical features of each patient were also noted. The presence of REM sleep disorders was determined by asking family members or caregivers. Brady- and akinesia, tremor, parkinsonian gait, and limb rigidity were considered parkinsonism findings. If at least one of these was present, the patient was considered to have parkinsonism.

### 2.4. Mortality status

Mortality during two-year follow-up was examined as the primary outcome of interest. We received death data from the Ministry of Health Death Registry File, which was supported by the information obtained from families and relatives.

### 2.5. Statistical analysis

All analyses were performed using the Statistical Package for Social Sciences (SPSS) version 16 (SPSS Inc., Chicago, IL, USA). Variables were given as absolute number and percentage, mean ± standard deviation, and median and interquartile range, as appropriate. The Mann-Whitney U test and Student’s t-test were used for continuous variables as appropriate. The chi-square test was used to compare categorical data. We performed univariate Cox regression analysis to assess the relationship between mortality and risk factors. Then, we built a multivariate Cox regression model to adjust for clinically and statistically significant variables. The following variables were considered for multivariable analysis: CDR (categorized as moderate-to-severe vs. mild), sex (female), age, functional impairment, and malnutrition. The results were reported as hazard ratios (HR) and 95% confidence interval (95%CI). A p-value of less than 0.05 was accepted as statistically significant.

## 3. Results

### 3.1. Baseline characteristics

A total of 108 patients with DLB (mean age: 78.9 ± 6.57 years) were included in the study. At the end of the 2-year follow-up period, 23 patients (21.3%) died, whereas 85 patients (78.7%) were alive. The majority of the participants were men (50.9%), less educated (≤5 years), married, and living with their spouses. The median CCI score was 5 (range, 3–8). Nonsurvivors had lower general cognitive performance (MMSE, p: 0.005; CDR, p: 0.008), more functional impairment (p: 0.001), and malnutrition (p: <0.001) than survivors. The other variables did not differ between the groups. Table shows the baseline demographics and disease characteristics of all patients according to their mortality status at the two-year follow-up.

### 3.2. DLB features

All participants had at least one type of core feature, whereas 88.9% had at least one type of supportive feature. The leading core feature was cognitive fluctuations (81.5%), followed by visual hallucinations (79.6%) and parkinsonism (74.1%). Among the supportive features, delusions (60.2%), other hallucinations (22.2%), and postural hypotension (4.6%) were the most common symptoms. Moreover, urinary incontinence was encountered in 60.6% of patients, and falls in 52.8% (Table).

### 3.3. Mortality risk factors

**Figure 2:**
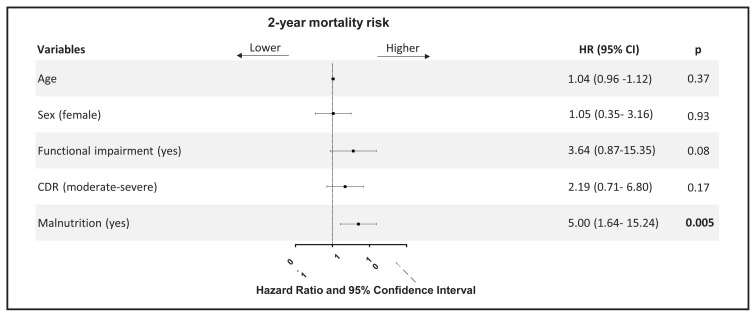
Forest plot graph of multivariate regression analysis of prognostic factors. Estimated hazard ratios were given with 95% confidence intervals. CDR: Clinical Dementia Rating.

We examined the risk factors for two-year all-cause mortality by using the Cox regression model. On the univariate Cox regression analysis, age (HR = 1.07; 95% CI: 0.995–1.156; p: 0.068), female sex (HR = 1.46; 95% CI: 0.57–3.7; p: 0.01), functional impairment (HR = 7.154; 95% CI: 1.98–25.88; p: 0.003), moderate to severe CDR (HR = 3.73; 95% CI: 1.43–9.73; p = 0.01), and malnutrition (HR = 6.54; 95% CI: 2.35–18.17; p: <0.001) were associated with the risk of two-year all-cause mortality. Other variables including MMSE, polypharmacy, visual hallucinations, cognitive fluctuations, parkinsonism, and REM sleep behavior disorder did not reach statistical significance in the univariate Cox regression analysis. On the multivariable analysis, only malnutrition (HR = 5.00; 95% CI: 1.64–15.24; p: 0.005) was an independent predictor of two-year all-cause mortality (Figure 2).

The Hosmer-Lemeshow (H-L) test, inferential goodness-of-fit test, yielded a chi-square of 17.611 and was insignificant (p: 0.854), suggesting that the model was a high fit of the data. The omnibus test confirmed that the model was highly significant (−2LL = 87.026, χ2(2) = 24.350, p: <0.001).

### 3.4. Sensitivity analysis

The sensitivity analysis did not change the results. After excluding five advanced dementia and five cancer patients (two prostate, one breast, one thyroid, one renal cell carcinoma), the association between malnutrition and 2-year all-cause mortality persisted after controlling for the variables (HR = 5.54; 95% CI: 1.72–17.78; p = 0.004).

## 4. Discussion

Predictors of mortality have been studied in DLB populations; however, from a clinical point of view, which is the aim of this study, no studies have attempted to determine mortality-related factors in these patients with special care needs. In our study, all-cause mortality risk was higher in female participants and in those with low cognitive status, functional impairment, and malnutrition. However, in multivariate regression analysis, malnutrition was found to be related to an increased risk of two-year all-cause mortality, probably due to altered immunity, reduced functional status, and worsening chronic conditions [24–26]. Our results highlight the need to assess nutritional status to identify older adults who may need nutritional interventions to reduce the risk of premature mortality in individuals with LBD. The present study also showed that DLB patients had a two-year all-cause mortality rate of 21.3%, suggesting a need for close medical follow-up and care in this population.

In dementia, the presence of malnutrition complicates the management of patients by increasing the risk of developing geriatric syndrome as well as impairing functionality, reducing quality of life, and leading to an increased risk of death [27]. In addition, the relationship between DLB and malnutrition was examined in two studies [27,28]. DLB patients had 6.83 times increased risk of malnutrition compared to those with other types of dementia [28]. Soysal et al. reported a malnutrition prevalence of 28.6% using the MNA-SF among patients with DLB, whereas in our study, the overall prevalence was 22.6%, which increased to 52.2% in the mortality group. The lower prevalence of malnutrition could be explained by the higher cognitive levels of our participants (mean MMSE score: 20.5 vs. 14.6). Moreover, two prospective cohorts, in which a decrease in BMI and lower hemoglobin and albumin levels, which are indicators of malnutrition, were detected during the course of DLB, partially supported our study [29,30]. No research has evaluated the relationship between malnutrition and mortality in patients with DLB. The current study showed that malnutrition was associated with 2-year all-cause mortality risk in this population. A recent study examining all types of dementia patients found that 31.4% of those with DLB had weight loss, but this factor only affected the emergency hospitalization risk, not mortality (HR = 0.94, %95 CI: 0.74–1.18) [27]. However, it is difficult to compare our study with these studies because the assessment of weight status alone may not be sufficient to reveal the nutritional status of an individual. As a result, nutritional screening could be integrated into the routine follow-up care of patients with DLB, as malnutrition may be a prognostic factor that increases the mortality risk. Further studies are needed to demonstrate possible effects of nutritional intervention on clinical outcomes in these vulnerable patients.

In our sample, we observed that female sex, advanced cognitive impairment, and functional impairment were associated with all-cause mortality within two years (not significant in the multivariate analysis) when considering the time elapsed from the clinical diagnosis of DLB to death. In individuals with these factors, the disease may progress rapidly, increasing the risk of death [31–34]. In a subgroup analysis of a study comparing DLB and AD patients in terms of mortality and survival, female DLB patients had shorter survival times than males after dementia diagnosis [14]. When patients were admitted to a nursing home, female DLB patients had a worse survival time than male patients [14]. Considering both these findings and those of our study, they should be interpreted with caution due to the small sample size. Thus, larger prospective studies are needed to examine mortality risk factors associated with DLB.

DLB patients after diagnosis have a shorter survival rate than those with AD and other dementia types [8,9,14,35]. The survival time ranges from 3.2 to 7.2 years [14,36,37]. Another important finding of the current study was that the all-cause mortality rate at two years, which was not examined in a previous study, was 21.3%. Although not directly comparable to our results, a cohort of 658 participants with DLB reported mortality rates of 12% in the first year and 76.3% in the fifth year [38]. Thus, these results demonstrate the short life expectancy of patients with DLB and warrant prospective studies and interventions to reduce early mortality in this under-studied population.

Our study had several limitations and strengths. Similar to any retrospective analysis, this research was limited to data already collected, which could be subject to selection bias and missing data. The retrospective design also limited our knowledge of the cause of mortality, which could not be directly related to dementia or related clinical parameters during the course of dementia. The study was single-center and did not include patients of different ethnic origins, which limited the generalizability of the study results. The monitoring period was relatively short; therefore, the effects of various factors on mortality could have been masked. The major strength of this study was the use of a large, well-defined dementia cohort established in a tertiary setting. Moreover, we evaluated the association between comorbidities and mortality using comprehensive clinical evaluation.

Given that patients with DLB have poorer prognosis than expected, it is critical to address coexisting comorbid health problems that complicate medical management and care. The findings of this study suggest that malnutrition may be an independent prognostic factor of long-term mortality in patients with DLB. Our study also increases awareness by showing that one out of every five people died within two years after DLB diagnosis. Identification of mortality risk factors can be an opportunity to prevent frequent hospital admissions, hospitalizations, and costs and ultimately, premature death in DLB patients. Further research with larger samples and longer follow-up periods is needed to confirm our findings.

## Figures and Tables

**Table:** Demographic and disease characteristics of the patient in terms of two-year mortality.

Variables	Total (n: 108)	Dead (n: 23)	Alive (n: 85)	p–value
Age, years, median*	79 (60–99)	82 (67–99)	79 (65–92)	0.10
Sex, female, n (%)	53 (49.1)	13 (56,5)	40 (47.1)	0.49
Education level, ≤5 years, n (%)	78 (72.2)	20 (87)	58 (68)	0.11
Marital status, married, n (%)	65 (60.2)	12 (52)	53 (62)	0.55
Living status, spouse, n (%)	62 (57.4)	9 (39.1)	53 (62.4)	0.07
Current smokers, n (%)	12 (11.1)	2 (8.7)	10 (11.8)	0.43
Current alcohol users, n (%)	2 (1.9)	0	2 (2.4)	1.00
CCI, median*	5 (3–8)	5 (4–8)	5 (3–8)	0.66
Cognitive status				
MMSE (0–30), median*	20.5 (3–26)	17 (3–25)	21 (9–26)	**0.005**
CDR (0–3), n (%)				
Mild	69 (63.9)	9 (39.1)	60 (70.6)	
Moderate	34 (31.5)	11 (47.8)	23 (27.1)	**0.008**
Severe	5 (4.6)	3 (13.0)	2 (2.4)	
Geriatric syndromes, n (%)				
Functional impairment	61 (56.5)	20 (87.0)	41 (48.2)	**0.001**
Urinary incontinence	65 (60.2)	15 (65.2)	50 (58.8)	0.63
Polypharmacy	63 (58.3)	13 (56.5)	50 (58.8)	1.00
Fall	57 (52.8)	13 (56.5)	44 (51.8)	0.82
Malnutrition a	24 (22.6)	12 (52.2)	12 (14.5)	**<0.001**
Core clinical features, n (%)				
Visual hallucinations	86 (79.6)	20 (87.0)	66 (77.6)	0.40
Cognitive fluctuations	88 (81.5)	18 (78.3)	70(82.4)	0.76
Parkinsonism	80 (74.1)	18 (78.3)	62 (72.9)	0.79
REM sleep behavior disorder	63 (58.3)	15 (65.2)	48 (56.5)	0.49
Supportive features, n (%)				
Delusions	65 (60.2)	15 (65.2)	50 (58.8)	0.64
Other hallucinations	24 (22.2)	6 (26.1)	18 (21.2)	0.59
Postural hypotension	5 (4.6)	0	5 (5.9)	0.36
Neuroleptic hypersensitivity	3 (2.8)	0	3 (3.5)	0.57

CCI: Charlson comorbidity index, CDR: Clinical Dementia Rating, MMSE: Mini–Mental State Examination, REM: rapid eye movement.

Values given in bold indicate statistically significant results (p < 0.05).

* Results were given with minimum and maximum values.

a Two missing data in malnutrition.
